# Depth-discrete metagenomics reveals the roles of microbes in biogeochemical cycling in the tropical freshwater Lake Tanganyika

**DOI:** 10.1038/s41396-021-00898-x

**Published:** 2021-02-09

**Authors:** Patricia Q. Tran, Samantha C. Bachand, Peter B. McIntyre, Benjamin M. Kraemer, Yvonne Vadeboncoeur, Ismael A. Kimirei, Rashid Tamatamah, Katherine D. McMahon, Karthik Anantharaman

**Affiliations:** 1 Department of Bacteriology, University of Wisconsin–Madison, Madison, WI USA; 2 Department of Integrative Biology, University of Wisconsin–Madison, Madison, WI USA; 3 Department of Natural Resources and the Environment, Cornell University, Ithaca, NY USA; 4 Department of Ecosystem Research, Leibniz Institute for Freshwater Ecology and Inland Fisheries, Berlin, Germany; 5 Department of Biological Sciences, Wright State University, Dayton, OH USA; 6 Tanzania Fisheries Research Institute (TAFIRI), Dar es Salaam, Tanzania; 7 Ministry of Livestock and Fisheries, Dodoma, Tanzania; 8 Department of Civil and Environmental Engineering, University of Wisconsin–Madison, Madison, WI USA

**Keywords:** Biogeochemistry, Limnology, Microbial ecology, Freshwater ecology

## Abstract

Lake Tanganyika (LT) is the largest tropical freshwater lake, and the largest body of anoxic freshwater on Earth’s surface. LT’s mixed oxygenated surface waters float atop a permanently anoxic layer and host rich animal biodiversity. However, little is known about microorganisms inhabiting LT’s 1470 meter deep water column and their contributions to nutrient cycling, which affect ecosystem-level function and productivity. Here, we applied genome-resolved metagenomics and environmental analyses to link specific taxa to key biogeochemical processes across a vertical depth gradient in LT. We reconstructed 523 unique metagenome-assembled genomes (MAGs) from 34 bacterial and archaeal phyla, including many rarely observed in freshwater lakes. We identified sharp contrasts in community composition and metabolic potential with an abundance of typical freshwater taxa in oxygenated mixed upper layers, and Archaea and uncultured Candidate Phyla in deep anoxic waters. Genomic capacity for nitrogen and sulfur cycling was abundant in MAGs recovered from anoxic waters, highlighting microbial contributions to the productive surface layers via recycling of upwelled nutrients, and greenhouse gases such as nitrous oxide. Overall, our study provides a blueprint for incorporation of aquatic microbial genomics in the representation of tropical freshwater lakes, especially in the context of ongoing climate change, which is predicted to bring increased stratification and anoxia to freshwater lakes.

## Introduction

Located in the East African Rift Valley, Lake Tanganyika (LT) holds 16% of the Earth’s freshwater and is the second-largest lake by volume. By its sheer size and magnitude, LT exerts a major influence on biogeochemical cycling on regional and global scales [1, 2]. For instance, LT stores over 23 Tg of methane below the oxycline [2], and about 14,000,000 Tg of carbon in its sediments [1]. Over the past centuries, LT’s rich animal biodiversity has been a model for the study of species radiation and evolution [3]. In contrast, the microbial communities in LT that drive much of the ecosystem-scale productivity remains largely unknown.

LT is over ten million years old and oligotrophic, which provides a unique ecosystem to study microbial diversity and function in freshwater lakes, specifically tropical lakes. The comparatively thin, oxygenated surface layer of this ancient, deep lake harbors some of the most spectacular fish species diversity on Earth [4], but surprisingly ~80% of the 1890 km^3^ of water is anoxic. Being meromictic, its water column is permanently stratified. This causes a large volume of anoxic and nutrient-rich bottom waters to be thermally isolated from the upper ~70 m of well-lit, nutrient-depleted surface waters. Despite stratification, periodically in response to sustained winds, pulses of phosphorus and nitrogen upwelling from deep waters replenish the oxygenated surface layers and sustain its productivity [5].

The physicochemical environment of LT is quite distinct from other ancient lakes [6, 7]. For example, Lake Baikal (LB), located in Siberia, is a seasonally ice-covered, of comparable depth (1642 m for LB, and 1470 m for LT), yet their thermal and oxygen profiles are drastically distinct. While LT is a permanently stratified layer with a large layer of anoxic waters, LB’s deepest layers are oxygenated. Previous work on LT’s microbial ecology documented spatial heterogeneity in microbial community composition, especially with depth [8, 9]. The observed differences were primarily related to thermal stratification, which leads to strong gradients in oxygen and nutrient concentrations. Early evidence from LT suggests that anerobic microbially driven nitrogen cycling such as anerobic ammonium oxidation (anammox) is an important component of nitrogen cycling [10]. However, the emergent effects of depth-specific variation in microbial communities on biogeochemical and nutrient cycling in LT remain largely unknown.

Here, we investigated microbial community composition, metabolic interactions, and microbial contributions to biogeochemical cycling along ecological gradients from high light, oxygenated surface waters to dark, oxygen-free and nutrient-rich bottom waters of LT. Our comprehensive analyses include genome-resolved metagenomics to reconstruct hundreds of bacterial and archaeal genomes, which were used for metabolic reconstructions at the resolution of individual organisms and the entire microbial community, and across different layers in the water column. In addition, we compared the microbial ecology in two contrasting ancient, and deep rift-formed lakes (LT and LB) to address questions about ecology, evolution, and endemism of microorganisms in freshwater lakes. Our work offers a window into the understudied microbial diversity of LT and serves as a case study for investigating microbial roles and links to biogeochemistry in globally distributed anoxic and deep freshwater lakes.

## Methods

### Sample collection

Samples were collected in LT, located in Central-East Africa, and based around the Kigoma and Mahale regions of Tanzania. We sampled near Kigoma because there are established field sites there and data that go back more than a decade. We sampled near Mahale National Park because of our focus on conservation in the nearly intact nearshore ecosystems there. LT has a strong latitudinal gradient (~673 km), therefore sampling in these two locations was also a way to capture some of that latitudinal variability.

**Fig. 1:**
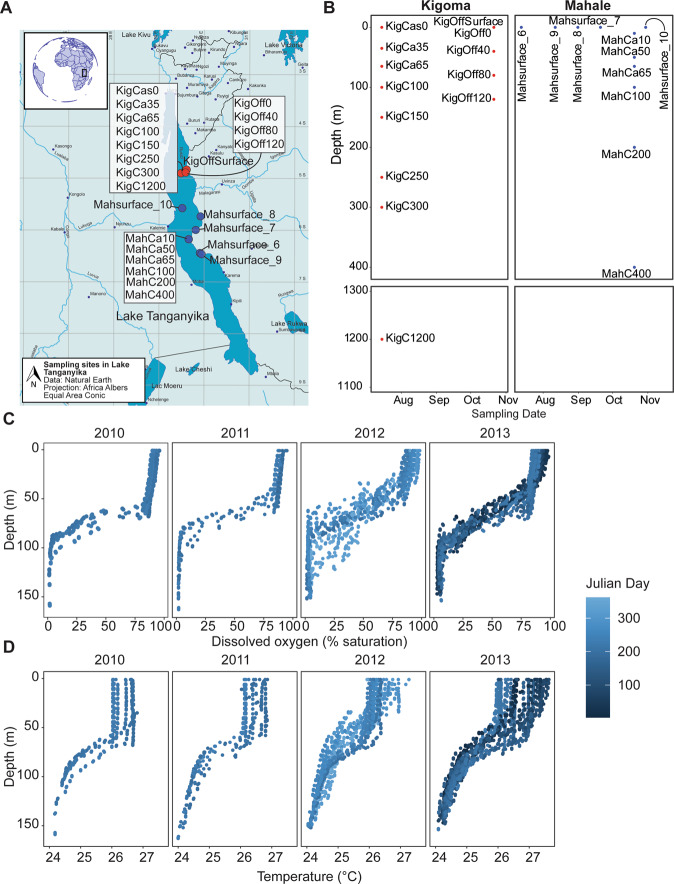
Sampling locations and environmental profiles in Lake Tanganyika. **A** Map of sampling locations in LT. The inset figure shows the approximate location of LT in a spherical global projection. Lakes and rivers are identified in blue; cities are shown in black. Rivers, lakes, and populous cities are labeled. The locations of the 24 samples collected are labeled, corresponding to Kigoma (red) or Mahale (blue), which are stations in Lake Tanganyika. The white shaded box represents location for which we collected depth-discrete samples (cast). **B** Sampling locations along the vertical transect of LT. Samples are depicted foremost by location (Kigoma vs. Mahale), followed by sampling date on the *x*-axis and depth on the *y*-axis. Short sample names are written next to each dot. **C** Dissolved oxygen (% saturation) in Lake Tanganyika, in percentage of saturation, from 2010 to 2013. **D** Temperature profiles, in degree Celsius, from 2010 to 2013 in Lake Tanganyika (Color figure online).

From 2010 to 2013, samples for chlorophyll a, conductivity, dissolved oxygen (DO), and temperature were collected. Secchi depths were collected over 2 years in 2012 and 2013, from a previous research cruise using a YSI 6600 sonde with optical DO and chlorophyll-a sensors. Those measurements were collected down to ~150 m. Twenty-four water samples were collected in 2015 for metagenome sequencing. A summary of all samples is available in Fig. S1. The samples were collected around two stations termed Kigoma and Mahale based on nearby cities. Water samples were collected with a vertically oriented Van Dorn bottle in 2015 and metadata is listed in Table S1. For the metagenomes, three Van Dorn bottle casts (10 L) in Kigoma and Mahale were collected, in which depth-discrete samples were collected across a vertical gradient, down to 1200 m at the maximum depth (Fig. 1 and Table S1). In addition, five surface samples were collected from the Mahale region, and one surface sample from the Kigoma region (Fig. 1). Methods to produce the map in Fig. 1 using the geographic information system ArcMap are described in the Supplementary Text.

We filtered as much as we could until the filter clogged, which was usually between 500 and 1000 mL. This usually took about 10–20 min of hand pumping. The water was not prefiltered, but filtered directly through a 47 mm 0.2-μm pore nitrocellulose filter, which was stored in a 2-mL tube with RNAlater. Filters were frozen immediately and brought back on dry ice to UW-Madison.

### DNA extraction and sequencing

DNA extractions were performed using the MP Biomedicals FastDNA Spin Kit with minor protocol modifications as described previously [11] back in Madison, WI. Metagenomic DNA was sequenced at the Joint Genome Institute (JGI) (Walnut Creek, CA) on the HiSeq 2500 platform (Illumina, San Diego, CA, U.S.A.), which produces 2 × 150 base pairs (bp) reads with a targeted insert size of ~240 bp.

### Metagenome assembly, genome binning, dereplication, and selection of genomes

Each of the 24 individual samples were assembled de novo by JGI to obtain 24 metagenomes assemblies. Briefly, raw metagenomic sequence reads were quality filtered, then assembled using MetaSPADEs v3.12 (default parameters) [12]. Each metagenome was binned individually using: MetaBat1 [13], MetaBat2 [14] v2.1.12, and MaxBin2 v2.2.4 [15]. We consolidated the bins generated by these three different tools using DASTool v1.1.0 [16] (default parameters except for --score_threshold 0.4), resulting in a total of 3948 MAGs. We dereplicated these MAGs using dRep v2.3.2 with the default settings [17]. A total of 821 unique clusters were created. Genome completion was estimated using CheckM v1.0.11 [18] and DAStool. To select a set of medium and high-quality genomes (≥50% completeness, <10% contamination) for downstream analyses, we used the minimum genome information standards for metagenome-assembled genomes [19], which yielded a total of unique dereplicated 523 MAGs (Table S2). In total, these representative MAGs represent clusters containing 3948 MAGs. The 523 MAGs are used as the dataset for this study.

### Relative abundance across water column depths and gene annotations

We mapped each metagenomic paired-read set to each of the 523 MAGs using BBMap [20], with default settings, to obtain a matrix of relative coverage (as a proxy for abundance across the samples) vs. MAG. We used pileup.sh implemented in BBMap, which calculates the coverage values, while also normalizing for the length of the scaffold and genome size [20]. We combined the mapping table from the 24 metagenomes, and summed the total coverage based on the associated MAGs identifier for each scaffold. To normalize the coverage by the metagenome size, we divided the coverage by the number of total reads per metagenome. Open reading frames of the scaffolds were identified using Prodigal v2.6.3 [21].

### Identification of phylogenetic markers

A set of curated Hidden Markov Models (HMM) for 16 single-copy ribosomal proteins (rpL2, rpL3, rpL4, rpL5, rpL6, rpL14, rpL14, rpL15, rpL16, rpL18, rpL22, rpL24, rpS3, rpS3, rpS8, rpS10, rpS17, rpS19) [22] was used to identify these genes in each MAG using hmmsearch (HMMER 3.1b2) [23] with the setting --cut_tc (using custom-derived trusted cutoffs). The esl-reformat.sh from hmmsearch was used to extract alignment hits.

### Phylogenetic tree

To create the concatenated gene phylogeny, we used reference MAGs, which represented a wide range of environments including marine, soil, hydrothermal environments, coastal and estuarine environments originally built from refs. [24, 25]. We used hmmsearch to identify the 16 ribosomal proteins for the bacterial tree, and 14 ribosomal proteins for the archaeal tree as described previously [24] (also described in “Identification of phylogenetic markers”). All identified ribosomal proteins for the backbone and the LT MAGs were imported to Geneious Prime V.2019.0.04 (https://www.geneious.com) separately for Bacteria and Archaea. For each ribosomal protein, we aligned the sequences using MAFFT (v7.388, with parameters: Automatic algorithm, BLOSUM62 scoring matrix, 1.53 gap open penalty, 0.123 offset value) [26]. Alignments were manually verified: in the case that more than one copy of the ribosomal protein was identified, we performed a sequence alignment of that protein using MAFFT (same settings) and compared the alignments for those copies. For example, if they corresponded exactly to a split protein, we concatenated them to obtain a full-length protein. If they were the same section (overlap) of the protein, but one was shorter than the other, the longer copy was retained. We applied a 50% gap masking threshold and concatenated the 16 (or 14) proteins. The concatenated alignment was exported into the fasta format and used as an input for RAxML-HPC, using the CIPRES server [27], with the following settings: datatype = protein, maximum-likelihood search = TRUE, no bfgs = FALSE, print br length = false, protein matrix spec: JTT, runtime = 168 h, use bootstrapping = TRUE. The resulting Newick format tree was visualized with FigTree (http://tree.bio.ed.ac.uk/software/figtree/). The same procedure was followed to create the taxon-specific tree, for example for the *Candidatus* Tanganyikabacteria and Nitrospira, using phylum-specific references except with no gap masking since sequences were highly similar and had few gaps. In addition, an *amoA* gene phylogeny was performed using UniProt *amoA* sequences with the two “predicted” comammox genomes with 90% masking (Supplementary Text).

### Taxonomic assignment and comparison of manual vs. automated methods

Taxonomic classification of MAGs was performed manually by careful inspection of the RP16 gene phylogeny, bootstrap values of each group, and closest named representatives (Supplementary Material 1 and 2). In addition, we also assigned taxonomy using GTDB-tk [28], which uses ANI comparisons to reference genomes and 120 and 122 marker genes for Bacteria and Archea, respectively, using FastTree (Supplementary Material 3 and 4). We compared the results for taxonomic classification between the manually curated and automated approaches to check whether taxonomic assignment matched across phyla, and finer levels of resolution. Since the two trees (RP16 and gtdb-tk tree) are made using different sets of reference genomes, we are unable to do a direct comparison of the taxonomic position of each of the MAGs in our study. Therefore, we manually curated each manual vs. GTDB-tk classification and added a column stating whether the results matched (Table S2).

To enhance the MAGs relevance to freshwater microbial ecologists, we assigned taxonomic identities to 16S rRNA genes identified in our MAGs to names in the guide on freshwater microbial taxonomy [29]. 16S rRNA genes were identified in 313 out of 523 MAGs using CheckM’s ssu_finder function [18]. The 16S rRNA genes were then used as an input in TaxAss [30], which is a tool to assign taxonomy to the identified 16S rRNA sequences against a freshwater-specific database (FreshTrain). This freshwater-specific database, albeit focused on epilimnia of temperate lakes, is useful for comparable terminology between the LT genomes and the “typical” freshwater bacterial clades, lineages and tribes terminology defined previously [29]. We also compared taxonomic identification among the three methods and show the results in Table S2.

We chose to provide the information from all sources of evidence (RP16, GTDB-tk tree, manual curation, automated taxonomic classification, and 16S rRNA sequences), even if in instances some results might be inconsistent. It is important to note that each source of evidence and reference-based methods are biased by their database content, but we hope that providing several lines of evidence can help arrive to a consensus. For readability, the MAGs in our study are referred by their manually curated phylum or lineage name.

### Support for Tanganyikabacteria, a monophyletic sister lineage to Sericytochromatia

We noted three MAGs from LT to be monophyletic, but initially were unrelated to any reference genomes. Since they were placed close to the Cyanobacteria, we created a detailed RP16 tree of 309 genomes of Cyanobacteria, and sister-lineages (Sericytochromatia/Melainabacteria [31], Blackallbacteria, WOR-1/Saganbacteria, and Margulisbacteria) including other freshwater lakes [32, 33], groundwater marine, sediment, fecal, isolates, and other environments. We calculated pairwise genome-level average nucleotide identities (ANI) values between all 309 vs. 309 genomes using fastANI [34]. The 3 genomes from LT were closely related, yet distinct from, the recently defined Cyanobacterial class Sericytochromatia [31, 35], using evidence from RP16 and GTDB-tk phylogeny of the MAGs, and ANI with closely related genomes in the literature (Table S3). The other existing Sericytochromatia used for comparison were isolated from Rifle acetate amendment columns (*Candidatus* Sericytochromatia bacterium S15B-MN24 RAAC_196; GCA 002083785.1) and a coal bed methane well (*Candidatus* Sericytochromatia bacterium S15B-MN24 CBMW_12; GCA 002083825)

### Comparison of MAG taxonomic diversity in LT vs. LB

To compare taxonomic diversity in two ancient deep lakes, LT and LB, we compare the ANI of metagenome-assembled genomes from the deepest samples from the two lakes. LB has 231 MAGs published [7]. These 231 MAGs were assembled from samples from depths of 1350 and 1250 m. We selected all MAGs that had a read abundance ≥0.5% of the KigC1200 sample (1200 m depth) microbial diversity, resulting in 260 MAGs. We used fastANI to compare all vs. all (491 vs. 491 MAGs) % ANI values. To assess the patterns, we generated histograms and mean ANI values and plotted them in R. We grouped the pairwise matches as Tanganyika vs. Tanganyika, Baikal vs. Baikal, and Tanganyika vs. Baikal (which is the same as Baikal vs. Tanganyika).

### Metabolic potential analysis and comparison of metabolic potential and connection across three distinct depths in LT

Metabolic potential of LT MAGs was assessed using METABOLIC, which includes 143 custom HMM profiles [36], using hmmsearch (HMMER 3.1b2) (--use_tc option) and esl-reformat to export the alignments for the HMM hits [23]. We classified the number of genes involved in metabolism of sulfur, hydrogen, methane, nitrogen, oxygen, C1-compounds, carbon monoxide, carbon dioxide (carbon fixation), organic nitrogen (urea), halogenated compounds, arsenic, selenium, nitriles, and metals. To determine if an organism could perform a metabolic function, one copy of each representative gene of the pathway must have been present in the MAG, for which a value of 1 (presence) was written, as opposed to 0 (absence). To investigate heterotrophy associated with utilization of complex carbohydrates, carbohydrate-degrading enzymes were annotated using hmmscan on the dbCAN2 [37] (dbCAN-HMMdb-V7 downloaded June 2019) database.

The sample profile collected on July 25, 2015 covered a vertical gradient ranging from surface samples to 1200 m. We analyzed samples collected near Kigoma from three different depths (0 m (KigCas0), 150 m (KigCas150), 1200 m (KigCas1200)) based on the general difference in abundance of key taxa. We used METABOLIC v4.0 [36] to identify and visualize organisms with genes involved in carbon, sulfur, or nitrogen metabolism. We combined the results into a single figure showing the number of genomes potentially involved in each reaction, and the community relative abundance of those organisms across three distinct depths. We performed some additional manual curation for differentiating between *amo* and *pmo* genes, and annotating *nxr* genes (Supplementary Text).

In addition to the functional gene annotations done by METABOLIC, which includes HMMs related to biogeochemical cycles, all 24 metagenomic assemblies were annotated by IMG/M, pipeline version 4.15.1 [38].

## Results

We sampled a range of physicochemical measurements in LT, which are described in Figs. 1 and S1. We collected 24 metagenome samples from the LT water column spanning 0–1200 m, at two stations Kigoma and Mahale in 2015 (Fig. 1B), which consisted of 18 depth-discrete samples and six surface samples. Despite not having paired physicochemical profiles in 2015, temperature and DO from 2010 to 2013 were highly consistent and showed minimal interannual variability, particularly during our sampling period (July and October) (Figs. 1 and S2). In addition, the environmental profiles are similar to those collected in other studies [39–42]. Water column temperatures ranged from 24 to 28 °C, and changes in DO were greatest at depths ranging from ~50 to 100 m during the time of sampling dropping to 0% saturation DO around 100 m. The thermocline depths shift vertically depending on the year (Fig. S2). Secchi depth, a measure of how deep light penetrates through the water column, was on average 12.2 m in July and 12.0 m in October (Fig. S3). Nitrate concentrations increased up to ~100 µg/L at 100 m deep, followed by rapid depletion with the onset of anoxia (Fig. S4). A consistent chlorophyll-a peak was detected at a depth of ~120 m in 2010–2013 (Fig. S5). Comparatively in 2018, the peak occurred around 50 m [42]. Other profiles collected in 2018 show a nitrate peak between 50 and 100 m in LT [42].

### The microbiome of Lake Tanganyika

**Fig. 2:**
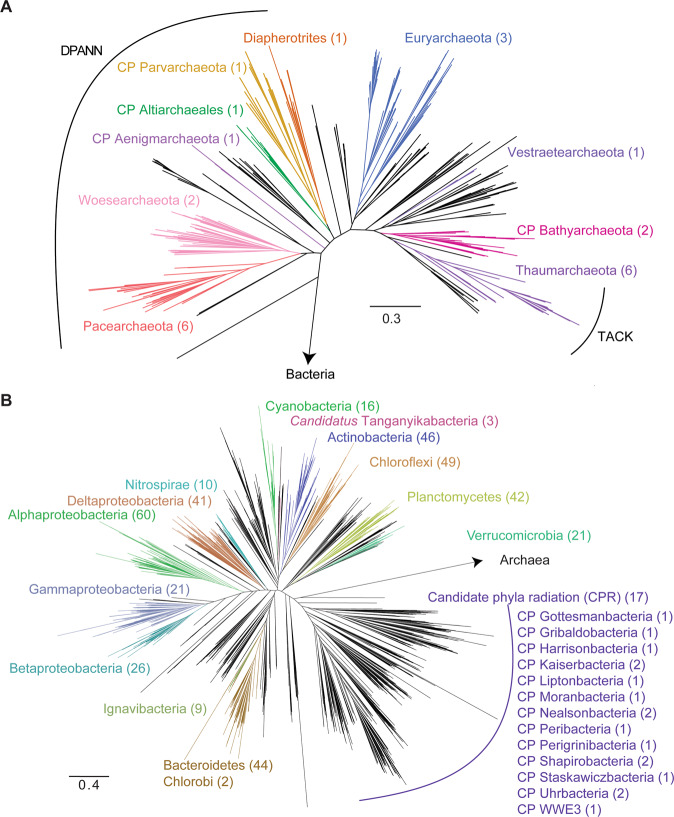
Phylogeny of Archaea and Bacteria identified in Lake Tanganyika. Phylogeny of **A** Archaeal and **B** Bacterial metagenome-assembled genomes (MAG) recovered from LT. The tree was constructed using 14 and 16 concatenated ribosomal proteins, respectively, and visualized using FigTree. Not all lineages are named on the figure. The groups DPANN (Diapherotrites, Parvarchaeota, Aenigmarchaeota, Nanoarchaeota, and Nanohaloarchaeota) and TACK (Thaumarchaeota, Aigarchaeota, Crenarchaeota, and Korarchaeota) archaea, CPR (candidate phyla radiation) bacteria are labeled. The number of medium- and high-quality MAGs belonging to each group are listed in parentheses. Colored groups represent the most abundant lineages in LT. Tanganyikabacteria MAGs from this study are italicized. A more detailed version of the tree constructed in iTol is found in Supplementary Material 1 and 2 with bootstrap values and names of taxa.

**Fig. 3:**
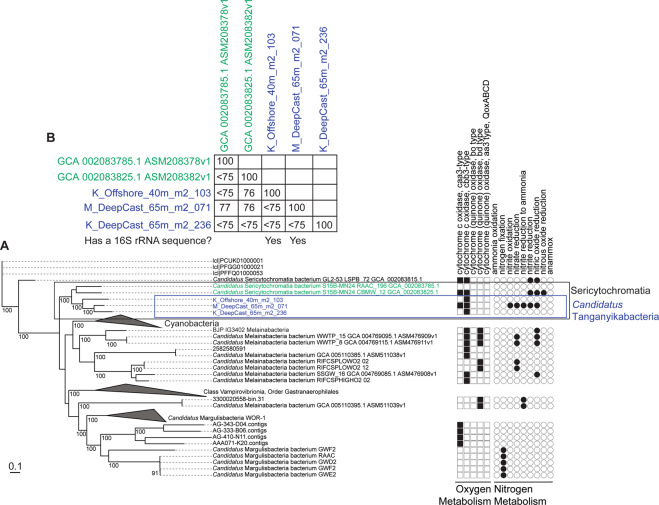
Concatenated gene phylogeny of Cyanobacteria and their non-photosynthetic sister-lineages, and comparison of average nucleotide identities. **A** Concatenated genome phylogeny using 16 ribosomal proteins of metagenome-assembled genomes of Cyanobacteria and non-photosynthetic sister-lineages such as Margulisbacteria (WOR-1), Melainabacteria, and Sericytochromatia. The three MAGs from Lake Tanganyika are labeled in blue, and represent a high-support (100) monophyletic lineage among the known Sericytochromatia. The presence–absence plot shows genes involved in oxygen metabolism (squares) and nitrogen metabolism (circles). The MAG from Lake Tanganyika is the only one among all genomes to have genes for denitrification. M_DeepCast_65m_m2_071 has ~97% genome completeness (full uncollapsed tree available in Supplementary Material). **B** Genome-level average nucleotide identity (ANI) (%) are shown as pairwise matrix for the five Sericytochromatia MAGs (boxed).

Metagenomic sequencing, assembly, and binning resulted in 3948 draft-quality MAGs that were dereplicated and quality-checked into a set of 523 non-redundant medium- to high-quality MAGs for downstream analyses. To assign taxonomic classifications to the organisms represented by the genomes, we combined two complimentary genome-based phylogenetic approaches: a manual RP16 approach, and GTDB-tk [28], an automated program which uses 120 concatenated protein coding genes. For the most part, we observed congruence between the two approaches (Fig. S6) and the genomes represented 24 Archaea and 499 Bacteria from 34 phyla, 74 classes, and 118 orders (Table S2 and Fig. 2). While manually curating the RP16 tree, and upon closer inspection and phylogenetic analysis of Cyanobacterial genomes and sister lineage genomes (Sericytochromatia, Melainabacteria, etc.), we noticed that three bacterial genomes formed a monophyletic freshwater-only clade within Sericytochromatia, sharing <75% genome-level ANI to other non-photosynthetic Cyanobacteria-like sequences (Fig. 3 and Table S3). On this basis, we propose to name this lineage *Candidatus* Tanganyikabacteria (named after the lake). Sericytochromatia is a class of non-photosynthetic Cyanobacteria-related organisms, that has recently gained attention along with related lineages such as Melainabacteria and Margulisbacteria due to the lack of photosynthesis genes, indicating phototrophy was not an ancestral feature of the Cyanobacteria phylum [31, 35]. This is the first recovery of Sericytochromatia from freshwater lake environments, since others were found in glacial surface ice, biofilm from a bioreactor, a coal bed methane well, and an acetate amendment column from the terrestrial subsurface.

**Fig. 4:**
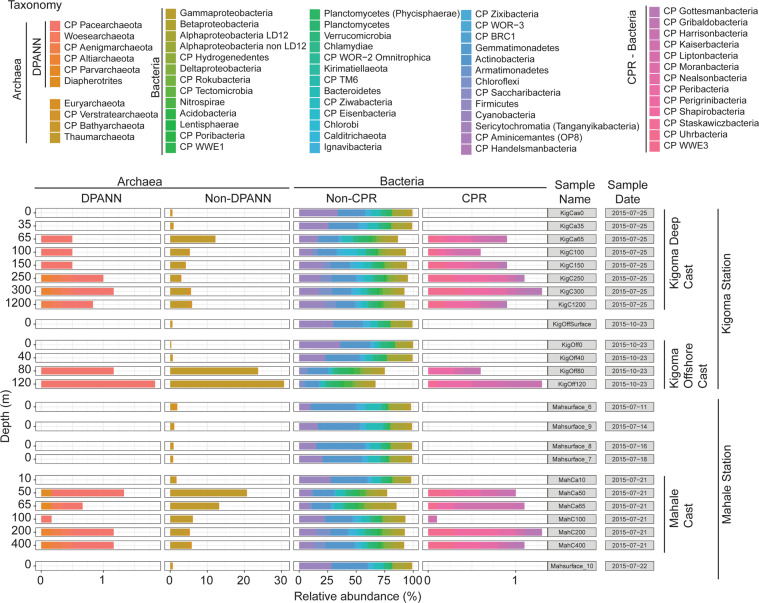
Bar plot showing the relative read abundance (RAR) per sample in the 24 metagenomes from Lake Tanganyika. MAGs are taxonomically ordered along the *x*-axis, with closely related taxa next to each other. In addition, information about whether they are Archaea (DPANN or not) and Bacteria (CPR or not) is specified. The three casts are identified with a vertical line on the right hand side. Samples are organized by location, then sampling date, then depth.

To investigate the stratification of microbial populations and metabolic processes in the water column of LT, we identified three zones based on oxygen saturation. The microbial community composition and community relative abundance (calculated as relative abundance of reads mapped, RAR) across our samples (Fig. S7) were distinct between the oxygenated upper layers and the deep anoxic layers (Fig. 4 and Table S4). Notably, Archaea accounted for up to 30% of RAR in sub-oxic samples (Kigoma 80 and 120 m), and generally increased in abundance with depth. DPANN archaea were only found in sub-oxic (>50 m deep) samples. Candidate phyla radiation (CPR) organisms generally increased in abundance with depth, reaching up to ~2.5% RAR. Among bacteria, notably, common freshwater taxa such as Actinobacteria (e.g., acI, acIV) Alphaproteobacteria (LD12), and Cyanobacteria showed a ubiquitous distribution, whereas certain groups such as Chlorobi and Thaumarchaetota were most abundant below 100 m. The community structure, composition, and abundance of the surface samples in the five Mahale samples were similar throughout. Note that the naming (e.g., K for Kigoma, M for Mahale, depths) of the LT MAGs originate from the sample they were originally binned (Table S1), however, it does not necessarily reflect that the MAG had the highest % RAR in that sample, since all representative MAGs are the result of dereplication (see “Methods”).

### How similar is the LT microbiome to that of other deep and ancient freshwater lakes?

**Fig. 5:**
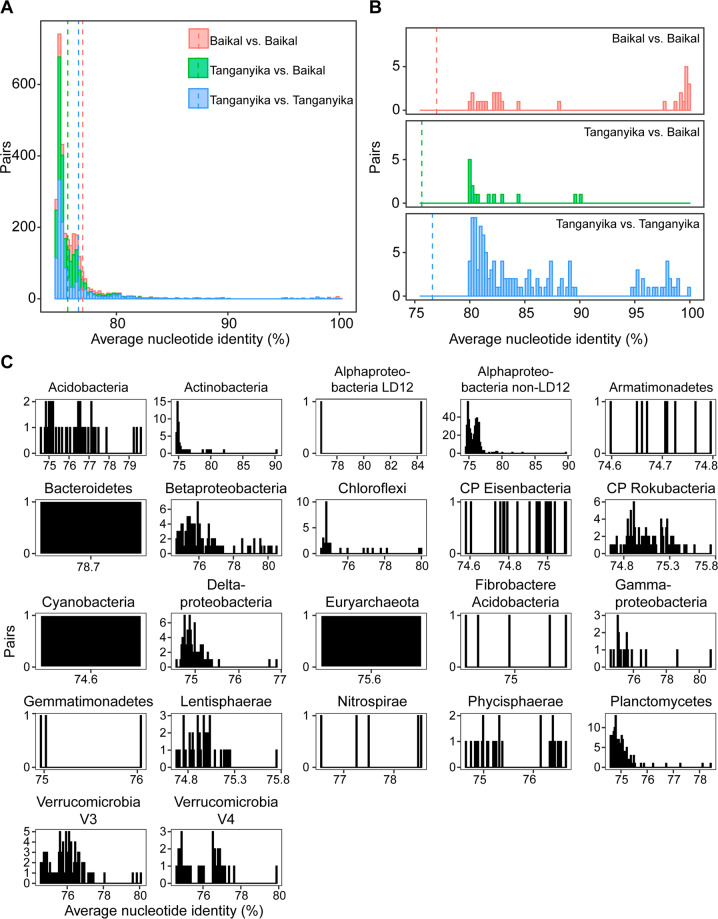
Comparison of metagenome-assembled genomes in Lake Tanganyika and Lake Baikal. **A** Histogram of the ANI values when doing pairwise comparison of full-genome MAGs from Lake Tanganyika vs. Lake Baikal. The vertical dashed lines correspond to the mean value (Baikal vs. Baikal: 79.98%, Tanganyika vs. Baikal: 75.63%, and Tanganyika vs. Tanganyika 76.60%). **B** Zoom in ANI values above 80% only. There are slightly more ANI above the 97% ANI for Baikal vs. Baikal, than Tanganyika vs. Tanganyika. Two genomes from Baikal had 100% ANI, two genomes from Tanganyika (K_Offshore_surface_m2_005 and K_DeepCast_100m_m2_150) shared 99.91% ANI. The highest % ANI between a MAG from Tanganyika vs. Baikal was among M_surface_10_m2_136 and GCA_009694405.1_ASM969440v1_genomic (90.19% ANI), which were *Candidatus* Nanopelagicaceae bacterium, an Actinobacteria. **C** Of the pairs in “Tanganyika vs. Baikal” category, showing the number of pairwise comparisons for each % ANI value, organized by taxonomic group. In **C**, all the *x*-axes are “% ANI.” For example, there is only one pair of Bacteroidetes, Cyanobacteria, and Euryarchaeota from the two lakes that have >75% ANI.

While a comparison of LT’s metagenomic diversity would be interesting to compare with other African Great Lakes (such as Lake Malawi, Lake Kivu, Lake Victoria), no published MAGs from those sites existed at the time of writing. We compared LT and LB’s microbial diversity (based on MAGs) because they are both ancient, extremely deep lakes, and therefore might both have sufficient evolutionary time for a wide diversity of bacterial and archaeal lineages to evolve. Yet, both lakes differ drastically in terms of environmental settings (LB is seasonally ice-covered with a oxygenated hypolimnion, whereas LT is a tropical ice-free lake with an anoxic bottom water layer). To compare the microbial communities, we compared the MAGs recovered from LT and LB (Figs. 5, S8 and Table S5).

Comparing the MAGs from the 1200 m sample in LT (KigOff1200, >0.05 % RAR) and MAGs from deep samples (1200 and 1350 m) from Baikal, relatively high RAR of Thaumarchaeota were identified in both lakes. Thaumarchaeaota accounted for ~10% RAR in LT and ~20% in LB. Typical freshwater taxa such as the acI lineage of Actinobacteria and LD12 group of Alphaproteobacteria (*Ca*. Fonsibacter)) were observed in higher abundance in surface samples but also at depths up to 50 m. CPR bacteria were found in both lakes. Archaea accounted for 6.3% of RAR in KigOff1200m, and about 2% in LB. Archaeal MAGs from KigOff1200 (and >0.05% RAR) belonged to the lineages Bathyarchaeota, Verstraetaerchaeota, Thaumarchaeota, Euryarchaeota, and DPANN (Pacearchaeota, Woesearchaeota, Aenigmarchaeota). The three DPANN MAGs from LB were closely related to Pacearchaeota and Woesearchaeota.

Interestingly, the majority of taxonomic groups observed in the deepest waters of LT (1200 m) was unique to LT (59%),whereas 68% of taxa richness recovered from LB that were bacterial MAGs were also identified in the deepest LT sample. Nitrososphaerales (Archaea) were identified in both LT and LB. Both LT and LB have a small abundance of Cyanobacteria. In LB, they account for ~1%, and are likely sourced from vertical mixing or sediment. However in LT, Cyanobacteria are more abundant, accounting for 14% RAR, but the lake is stratified with no mixing. Organisms from the lineage Desulfobacterota, with prominent roles in sulfur cycling (H_2_S generation) were identified in LT but not in LB. Overall, both lakes had high abundance and richness of Archaea and CPR, although the specific linneages (for example Desulfubacterota) likely reflect the differences in geochemistry in the lake, and to the different oxygen niches that these organisms may occupy.

### Depth-dependent contrasts in microbial metabolism in LT: biogeochemical cycling of carbon, nitrogen, and sulfur

**Fig. 6:**
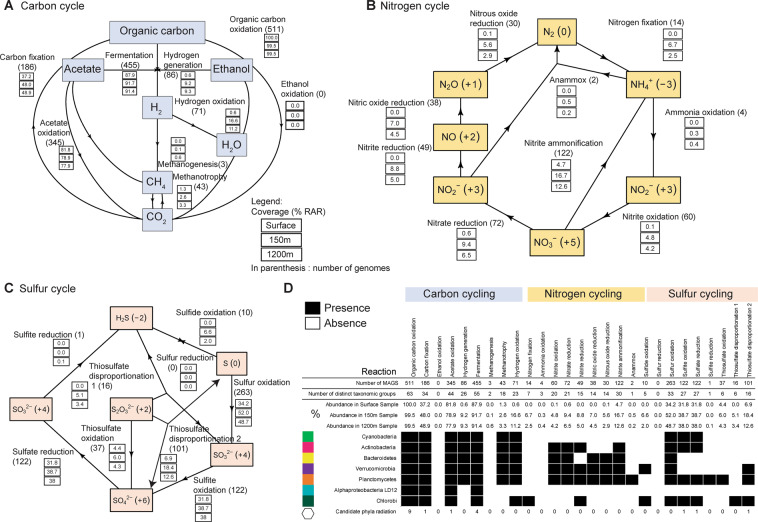
Microbial contributions to carbon, nitrogen and sulfur cycling in Lake Tanganyika. Abundance of organisms involved in different **A** carbon, **B** nitrogen, and **C** sulfur cycling steps, in three depth-discrete samples from Kigoma (surface, 150 m, 1200 m).Oxidation states are shown in parentheses. **D** Presence and absence of individual steps in C, N, S cycling. Abundance of organisms is described in percentages. Only reactions in a subset of common freshwater taxa (Cyanobacteria, Actinobacteria, Bacteroidetes, Verrucomicrobia, Planctomycetes, Alphaproteobacteria (LD12), Chlorobi) and candidate phyla radiation are shown. Presence and absence of these pathways in taxonomic groups are represented by filled and open circles, respectively.

We investigated microbial metabolic potential and process-level linkages between MAGs in the surface, at 150 m, and at 1200 m, representing three distinct ecological layers within the lake (Fig. 6 and Tables S6, 7). Individual metabolic pathways were identified in each MAG and their capacity to contribute to different (Fig. S9 and Tables S6, 7), nitrogen (Fig. S10 and Table S6, 7), and sulfur (Fig. S11 and Table S6, 7) biogeochemical cycles were assessed (Fig. S12 and Table S6, 7). Based on historical environmental data [39], light sufficient to support photosynthesis is available to ~70 m, and these surface waters are oxygen-rich and nutrient-depleted. The 150 m depth is subject to more interannual year variation in temperature and DO (Figs. S2–5). The 1200 m sample represents a relatively stable, dark, nutrient-rich, and anoxic environment, according to data collected from 2010 to 2013 in our study and in accordance with historical profiles.

Microbial pathways associated with the carbon cycle included the use of organic carbon (e.g., sugars), ethanol, acetate, methanogenesis, methanotrophy, and CO_2_-fixation (Fig. 6). As would be expected, organisms capable of organic carbon oxidation were abundant comprising nearly 99% RAR community in the sub-oxic and anoxic samples. The metabolic potential for fermentation and hydrogen generation was also abundant in the sub-oxic and anoxic samples, represented by 91% RAR (455 MAGs) and 9% RAR (86 MAGs), respectively. Other anerobic processes including methanogenesis (3 MAGs) and methanotrophy (43 MAGs) were identified in a limited number of MAGs, but were observed to be more abundant in the 1200 m samples. Both bacteria and archaea encoded carbohydrate-degrading enzymes (CAZYmes) (Fig. S13 and Table S8). The highest densities of CAZYmes (when normalized by genome size) were identified in organisms from the lineages Verrucomicrobia, Lentisphaerae, and CP Shapirobacteria. Meanwhile, glycoside hydrolases (GHs) that participate in the breakdown of different complex carbohydrates were prominent in Verrucomicrobia and Planctomycetes, as has been observed in other studies of these common freshwater linneages in lakes [43, 44].

We identified microorganisms involved in the inorganic nitrogen cycle, including oxidation and reduction processes (Fig. 6 and Table S6, 7). The metabolic potential for nitrogen cycling was generally higher in the sub-oxic samples as compared to the anoxic samples. Nitrogen fixation capacity (14 MAGs) was found below the surface, and nitrogen fixing organisms comprised up to 7% RAR at each depth. We identified a MAG belonging to the Thaumarchaeal class Nitrososphaeria that contains Archaeal *amoABC* genes for ammonia oxidation (Supplementary Material 5), and many non-CPR Bacteria with *nxr* genes involved in nitrite oxidation (Fig. S14). Nitrospira bacteria were identified to be capable of complete ammonia oxidation (comammox) (Clade II-A), with highest RAR in the sub-oxic depths (Fig. S15). Denitrification processes that remove nitrate and nitrite from the system were discovered across all depths, at approximately the same RAR in all depths. For example, the potential for nitrate reduction was identified in 72 MAGs that comprised 6.5–9% RAR and for nitrite reduction in 49 MAGs, at 5–9% RAR in anoxic depths. The nitrate/nitrite ammonification (DNRA) potential was identified in 122 MAGs accounting for between 5 and 17% RAR in each depth. Bacteria capable of anammox accounted for between 0.2 and 5% RAR at each depth.

Sulfur biogeochemical profiles exist in LT and generally show an increase in H_2_S beginning in the sub-oxic zone into the anoxic zone with the highest concentration observed in the deepest waters [39]. H_2_S can accumulate in the water column through sulfite reduction, thiosulfate disproportionation, and sulfur reduction. While only a few MAGs were potentially able to perform these processes (1, 16, and 10, respectively), we observed that all of them increased in RAR with depth and were more abundant in the anoxic depths (Fig. 6). Sulfur oxidation (263 MAGs), sulfite oxidation (122 MAGs), and sulfate reduction were each prominently represented processes, with such organisms accounting for over 30% RAR at each depth.

To understand the behaviors of carbon, sulfur, and nitrogen biogeochemical cycles in the LT water column, we investigated the depth distribution of different microbially mediated processes. We observed differences amongst the carbon, sulfur, and nitrogen cycles. Processes that were more prominent at sub-oxic and anoxic depths, but still present at low (<5% RAR) from the surface include nitrogen cycling (nitrite oxidation, nitrous oxide reduction, nitrite ammonification, nitrate reduction) and carbon cycling (methanotrophy, hydrogen oxidation, and hydrogen generation). Processes that were exclusive to the sub-oxic and anoxic depths include carbon cycling (methanogenesis), nitrogen cycling (nitrogen fixation, ammonia oxidation, nitrite oxide reduction, nitric oxide reduction, anammox), and sulfur cycling (sulfide oxidation, sulfite reduction, thiosulfate disproportionation H_2_S and SO_3_^2−^). The potential for use of alternative electron acceptors such as chlorate, metals, arsenate, and selenite was also identified in organisms in sub-oxic and anoxic depths.

**Fig. 7:**
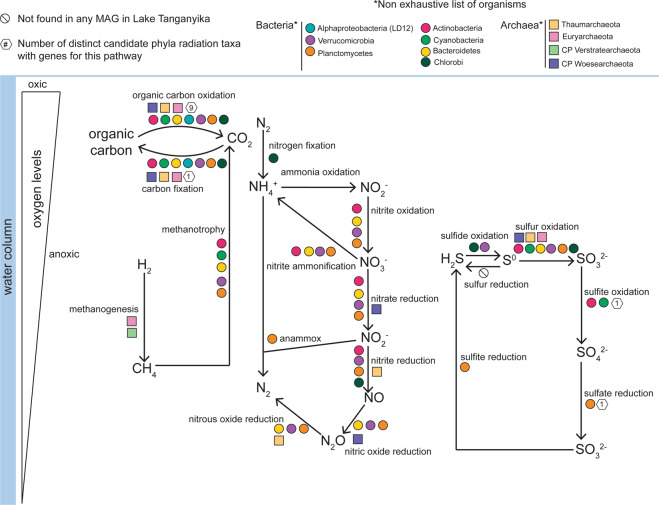
Summary figure showing the role of different microbial taxa in carbon, nitrogen, and sulfur cycling in Lake Tanganyika. The list of organisms shown and the reactions are not exhaustive.

Given our earlier finding that the water column was populated with common freshwater taxa in the upper mixed layer, vs. less common organisms in the anoxic water column, we wanted to know if the differences were reflected in metabolism of carbon, nitrogen, and sulfur across these depths. We identified the metabolic potential of organisms from the lineages Cyanobacteria, Actinobacteria, Alphaproteobacteria (LD12), Verrucomicrobia, Planctomycetes, and Bacteroidetes (Fig. 6). We counted the number of distinct phylogenetic taxa with the potential to conduct a biogeochemical transformation. The potential for nitrogen fixation was not identified in Cyanobacteria MAGs from the water column. The Cyanobacteria in LT were all identified as belonging to Synechoccales, which are known for not being nitrogen fixers [45–48]. Instead, a small group of microorganisms from seven distinct taxa, which were mostly abundant at sub-oxic and anoxic depths, was observed to be capable of nitrogen fixation (Deltaproteobacteria, Kirimatiellacea, Alphaproteobacteria (non-LD12), Chloroflexi, Chlorobi, Euryarchaeota, Gammaproteobacteria). The inferred involvement of microbes in biogeochemical cycling across an oxygen gradient such as in LT is summarized in Fig. 7.

## Discussion

The natural history of LT, along with its environmental characteristics, makes it ideal for examining the role of microbial communities in nutrient and biogeochemical cycling in tropical freshwater lakes. Being an ancient, deep, stratified, and meromictic lake with varying oxygen concentrations, it also serves as a case study for microbial diversity and metabolism in permanently anoxic environments. Here we highlight that microbial diversity and function in LT is distinct between its oxic mixed surface waters, and its anoxic deep waters. We note that although MAGs have key genes to participate in a given process, this does not necessarily mean that the corresponding organisms are actively using them all the time. As such, the purpose of our study was to provide an overview of the metabolic potential for microbially driven biogeochemical processes in LT and to guide future hypotheses for the study of tropical freshwater lakes under stress from a changing climate.

### Comparison of microbiomes between two deep rift valley lakes, LT and Baikal: endemism vs. shared lineages

LT (1470 m) and LB (1642 m) are the world’s two deepest freshwater lakes, yet are drastically different in their water column characteristics. In addition, both lakes are ancient lakes: LT is approximately ten million years, and LB is 25 million years. Since few metagenomic studies have been conducted on deep freshwater lakes, a comparison of LT and LB is informative in addressing questions about ancient microbial lineages, evolution, and endemic or shared lineages. As mentioned previously, LT is tropical and has an anoxic bottom layer. LB is located in Siberia, is seasonally ice-covered (twice a year), is dimictic (mixed twice a year), and has an oxygenated bottom layer. Both are ancient and formed geologically through rifts. LB’s microbial community has been studied using metagenomics, from depths ranging from 0 to 70 m during the ice-cover period [6, 49] and from 1250 to 1300 m deep collected in March 2018 also during an ice-covered period [7]. LT’s microbiome has been studied via 16S rRNA amplicon sequencing in the past [8–10] and in this study via metagenomics. While LT is primarily anoxic below ~100–50 m, LB has deep-water currents near the coastal regions, which results in oxygenation of deep waters. The regular mixing from surface waters from LB may imply lower endemism, as the “deep-water” ecological niche would be more frequently disturbed than in meromictic LT.

On one hand, because both LT and LB are ancient, deep, and rift valley freshwater lakes, we hypothesized that they might have a core microbiome common to both (shared lineages). However, given their contrasting environments (cold vs. tropical, oxic vs. anoxic), we expected that environmental characteristics driven by oxygen presence would be major drivers of microbial community structure and function, such as is the case in soils [50], sediments [51], marine oxygen-minimum zones [52] rather than ecosystem type, especially with the increased importance of nitrogen and sulfur cycling under low-oxygen conditions. In our study for example, we find taxa such as sulfur cycling Desulfobacterota only in LT and not in Baikal, likely reflecting that these H_2_S producers are found in low-oxygen environments.

### Metabolic connections across vertical gradients in LT

Our metabolic function analyses show the distribution of microbially driven carbon, nitrogen, and sulfur cycling across a depth gradient in LT. The carbon cycle of LT has mostly been investigated from the perspective of primary production, and methane cycling. It has been suggested that anaerobic methane oxidation contributes to decreasing the amount of methane in the anoxic zone of LT [2]. Methane is a greenhouse gas that is important in LT, particularly with respect to stratification. Because methane is more abundant below the oxycline, a shift in the oxycline could lead to methane release to the atmosphere via ebullition. Bacterial activity is thought to be a source of methane in nearby (~470 km away) Lake Kivu [53]. Major findings in methanogenesis in aquatic ecosystems now bring light to previously unrecognized biological sources and sinks of methane [54, 55]. We and others [2] have found methanogenic Archaea in LT.

Nitrogen cycling has been well-studied in LT, mostly focused on nitrogen inputs and outputs, and from a chemical and biogeochemical perspective. For example, atmospheric deposition and nitrogen from river inflow contribute much of the nitrogen to LT [56]. In addition, it is shown that incoming nitrogen in the form of nitrate is quickly used by organisms in the surface waters, and the upper water column is often nitrate depleted. Profiles of nitrogen (NH_4_^+^, NO_2_^−^, NO_3_^−^) in LT show that NH_3_ accumulated from 200 to 1200 m, NO_2_^−^ is usually overall low throughout the water column, and NO_3_^−^ is low at the surface, peaks at the oxycline, and declines where NH_4_^+^ increases [39]. Another way that NH_4_^+^ can be supplemented to the water column is through nitrogen fixation. In freshwater systems, it is typically thought that this process is conducted by Cyanobacteria, particularly the heterocystous *Anabaena flos-aquae* [57]. In Lake Mendota, a eutrophic temperate freshwater lake, metagenomic data hAVE shown that a third of nitrogen fixation genes originate from Cyanobacteria, and the remaining from Betaproteobacteria and Gammaproteobacteria [43]. However, we were surprised to find that nitrogen fixation was not identified in any LT Cyanobacteria MAGs from the water column. Benthic nitrogen fixers (cyanobacteria and diatoms with endosymbionts) are dominant in the littoral zone of LT and can provide as much as 30% of N in nearby lake Malawi [58]. Rather, from the metagenomic data of free-living planktonic bacteria, nitrogen fixation was identified to potentially be perfomed in other groups such as Deltaproteobacteria, Kirimatiellacea, Alphaproteobacteria, Chloroflexi, Chlorobi, Euryarchaeota, and Gammaproteobacteria. Similar results have been observed in Trout Bog Lake, a stratified humic lake where the nitrogen fixers were more diverse and also included Chlorobi [43], like in LT. From the chemical evidence, we hypothesized that the oxycline is a hotspot for nitrite oxidation, and denitrification occurs below the oxycline. Nitrite oxidation pathways in LT MAGs show that many organisms are potentially able to perform the reaction; however, the *nxr* enzyme may act reversibly (NO_2_^−^ to NO_3_^−^ and vice-versa). Denitrification processes (nitrate reduction, nitrite reduction, nitric oxide reduction, and nitrous oxide reduction) have similar representation across all three depths. A study of nitrogen cycling processes in LT [59] identified very low dissolved inorganic nitrogen concentrations in the euphotic zone, and proposed that very active N cycling must be occurring in the water column to prevent this accumulation. Another process leading to low dissolved inorganic nitrogen concentration is anammox. Incubations with ^15^N-labeled nitrate have showed that anammox was active in the 100–110 m water depth, and was comparable to rates observed in marine oxygen-minimum zones [10]. Our detection of Planctomycetes bacteria capable of anammox (Brocadiales) supports this conclusion.

Sulfur cycling in freshwater lakes has received less attention compared to carbon, nitrogen, and phosphorus. Profiles from LT show a clear increase in H_2_S below the oxycline, stabilizing at a concentration of ~30-μM H_2_S from 300 to 1200 m depth [39]. Biologically, H_2_S is produced via sulfite reduction, thiosulfate disproportionation, sulfur reduction, all of which were identified exclusively in the sub-oxic and anoxic depths. Given the use of sulfur-based electron acceptors as alternatives to oxygen in anoxic systems, sulfur cycling has been documented to increase in importance with lower-oxygen availability. As one of the critical compounds for life, sulfur availability and processing are essential to support cellular functions. In LT, where a large portion of the water column is anoxic, sulfur cycling is expected to support primary productivity in the upper water column, and have an overall important effect on lake ecology and biogeochemistry [60]. While the abundance of sulfur cycling organisms suggests an active sulfur cycle in LT, intriguingly, the abundance of organisms mediating the oxidation of reduced sulfur compounds (H_2_S, S^0^, S_2_O_3_^2−^) far exceeded the abundance of organisms mediating the reduction of sulfur compounds (SO_3_^2−^, SO_4_^2−^, S^0^). While this discrepancy may arise from increased enzymatic activity of sulfate/sulfite and sulfur reducing organisms, it may also be associated with the geochemistry of the deep waters in LT. Being a rift valley lake, it is home to hydrothermal vents, in which reduced compounds and gases from the Earth’s crust including H_2_, H_2_S, and CH_4_ may be produced [61, 62]. Around these hydrothermal vents, white and brown microbials mats have previously been identified [62] and microbial life is abundant. [63]. In marine environments, *Beggiatoa* mats are associated with sulfide oxidation [64], and Zetaproteobacteria are often the primary iron oxidizers in iron-rich systems, creating brown mats [65].

## Conclusions

Our study provides genomic evidence for the capabilities of Bacteria and Archaea in tropical freshwater biogeochemical cycling and describes links between spatial distribution of organisms and biogeochemical processes. These processes are known to impact critical food webs that are renowned for their high biodiversity and that serve as important protein sources for local human populations, as the shoreline of LT is shared by four African countries. As a permanently stratified lake, LT also offers a window into the ecology and evolution of microorganisms in tropical freshwater environments. Our study encompassed the lake’s continuous vertical redox gradient, and shows that microbial communities were dominated by core freshwater taxa at the surface and by relatively high abundances of Archaea and uncultured candidate phyla in the deeper anoxic waters compared with freshwater lake counterparts. The high prominence of Archaea making up to 30% of the community composition abundance at lower depths further highlighted this contrast. While freshwater lakes are often limited by phosphorus [66], tropical freshwater lakes are frequently nitrogen-limited [67]. Our microbe-centric analyses reveal that abundant microbes in LT play important roles in nitrogen transformations thay may remove fixed nitrogen from the water column, or fix nitrogen that may be upwelled and replenish the productive surface waters in bioavailable forms of fixed nitrogen.

Tropical lakes are abundant on earth, yet understudied both in the limnological literature and in the context of microbial metabolism and biogeochemistry. Yet, it is critically important to understand the biogeochemistry of tropical lakes, as lake ecosystem health is critical to the livelihood of millions of people, for example in LT [68, 69]. With increasing global temperatures, extremely deep lakes such as LT will likely experience increased stratification, lower mixing, and increased anoxia [70]. We provide the most comprehensive metagenomics-based genomic study to date of microbial community metabolism in an anoxic tropical freshwater lake, which is an essential foundation for future work on environmental adaptation, food webs, and nutrient cycling. Overall, our study will enable the continued integration of aquatic ecology, “omics” data, and biogeochemistry in freshwater lakes, that is key to a holistic understanding of ongoing global change and its impact on surface freshwater resources.

## Supplementary information

Supplementary Text

Figure S1

Figure S2

Figure S3

Figure S4

Figure S5

Figure S6

Figure S7

Figure S8

Figure S9

Figure S10

Figure S11

Figure S12

Figure S13

Figure S14

Figure S15

Supplementary Material 1

Supplementary Material 2

Supplementary Material 3

Supplementary Material 4

Supplementary Material 5

Table S1

Table S2

Table S3

Table S4

Table S5

Table S6

Table S7

Table S8

## Data Availability

The MAGs can be accessed on NCBI BioProject ID PRJNA523022. The individual accession IDs for each genome are in Table S2. The genomes will be officially released on NCBI Genbank upon publication. All MAGs are also publicly available on the Open Science Framework: https://osf.io/pmhae/. The 24 raw, assembled, and annotated metagenomes are available on the Integrated Microbial Genomes & Microbiomes (IMG/M) portal using the following IMG Genome IDs: 3300020220, 3300020083, 3300020183, 3300020200, 3300021376, 3300021093, 3300021091, 3300020109, 3300020074, 3300021092, 3300021424, 3300020179, 3300020193, 3300020204, 3300020221, 3300020196, 3300020190, 3300020197, 3300020222, 3300020214, 3300020084, 3300020198, 3300020603, 3300020578. An interactive version of the Archaeal and Bacteria trees can be accessed at iTOL at: https://itol.embl.de/shared/patriciatran. Code to generate the figures, and access to the custom HMM for the 16 ribosomal proteins and the metabolic genes is available at https://github.com/patriciatran/LakeTanganyika/.
