# Supplementary material for: Depth-discrete metagenomics reveals the roles of microbes in biogeochemical cycling in the tropical freshwater Lake Tanganyika
Source: ISME J. 2021 Feb 9;15(7):1971–86. doi: 10.1038/s41396-021-00898-x (PMC8245535; doi:10.1038/s41396-021-00898-x)
Supplement: Supplementary file 2 — Figure S1 [file 41396_2021_898_MOESM2_ESM.pdf]

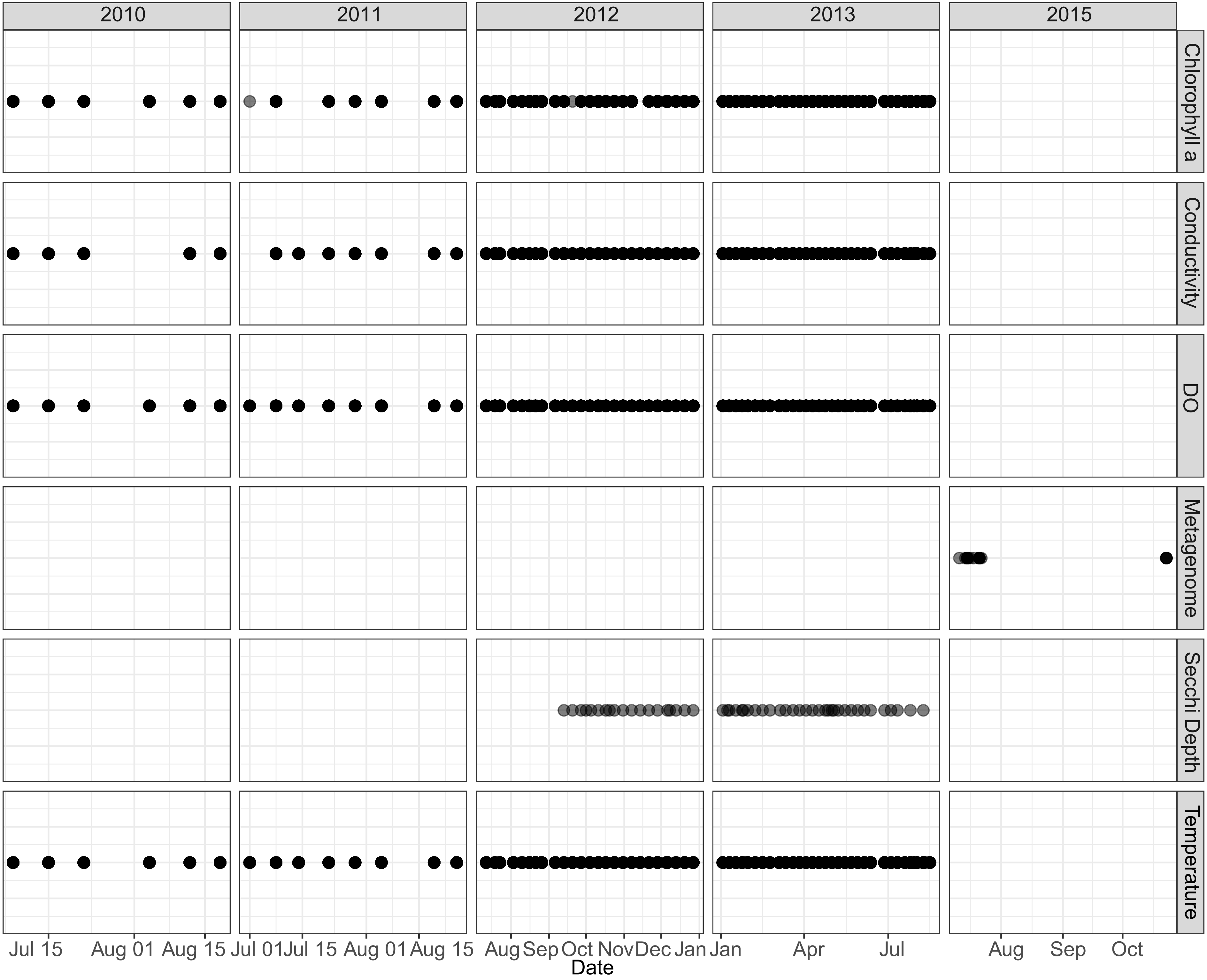

Supplementary Figure 1. Overview of all samples from this study. Chlorophyll a, conductivity, dissolved oxygen (DO), and temperature was collected from 2010 to 2013. Secchi depth data are available from 2012 and 2013. Metagenome samples were collected in 2015.
