# Supplementary material for: Depth-discrete metagenomics reveals the roles of microbes in biogeochemical cycling in the tropical freshwater Lake Tanganyika
Source: ISME J. 2021 Feb 9;15(7):1971–86. doi: 10.1038/s41396-021-00898-x (PMC8245535; doi:10.1038/s41396-021-00898-x)
Supplement: Supplementary file 3 — Figure S2 [file 41396_2021_898_MOESM3_ESM.pdf]

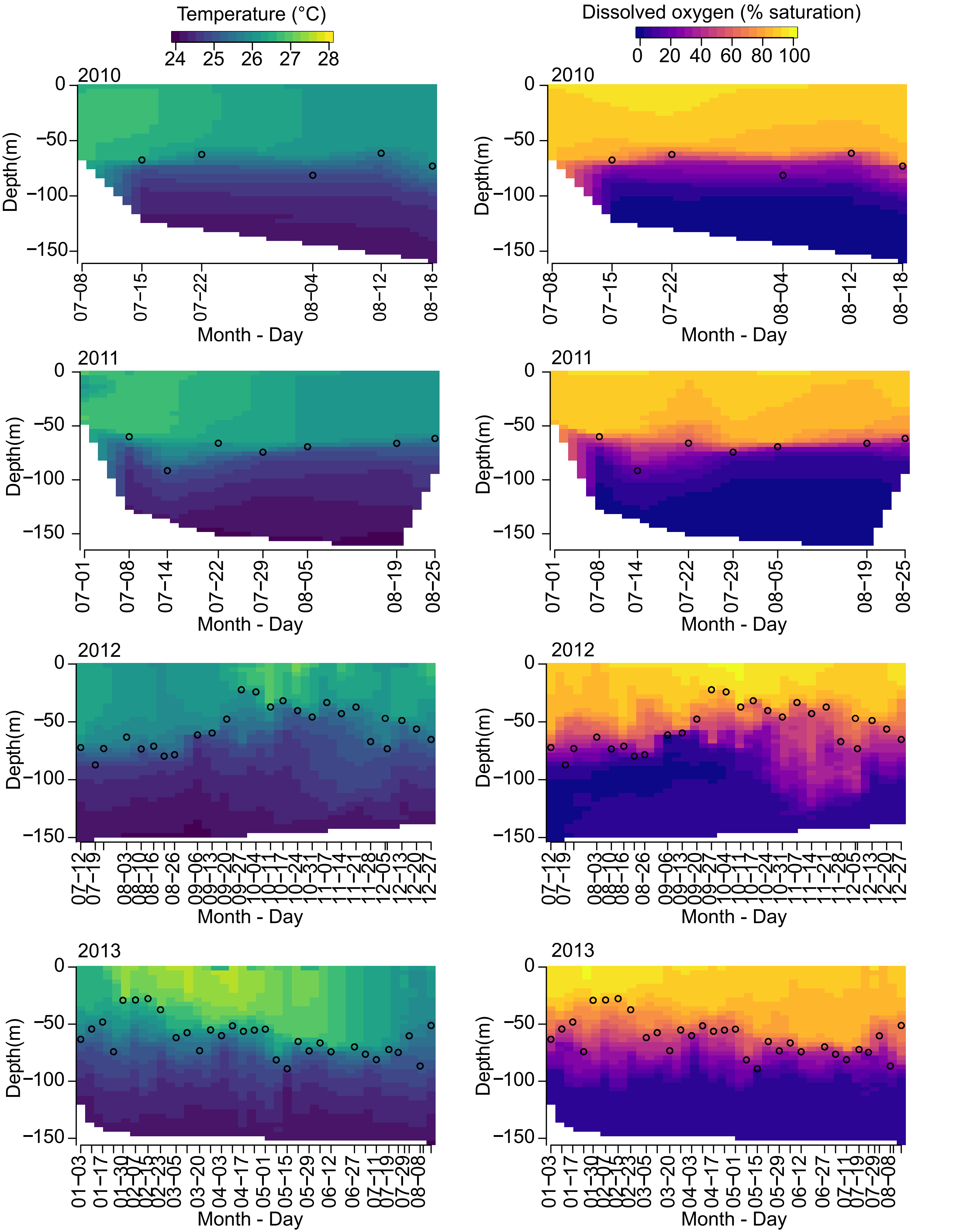

Figure S2. Environmental data profiles from 2010-2013. Dots represent the thermocline depth at each sampling date.
