# Supplementary material for: Depth-discrete metagenomics reveals the roles of microbes in biogeochemical cycling in the tropical freshwater Lake Tanganyika
Source: ISME J. 2021 Feb 9;15(7):1971–86. doi: 10.1038/s41396-021-00898-x (PMC8245535; doi:10.1038/s41396-021-00898-x)
Supplement: Supplementary file 6 — Figure S5 [file 41396_2021_898_MOESM6_ESM.pdf]

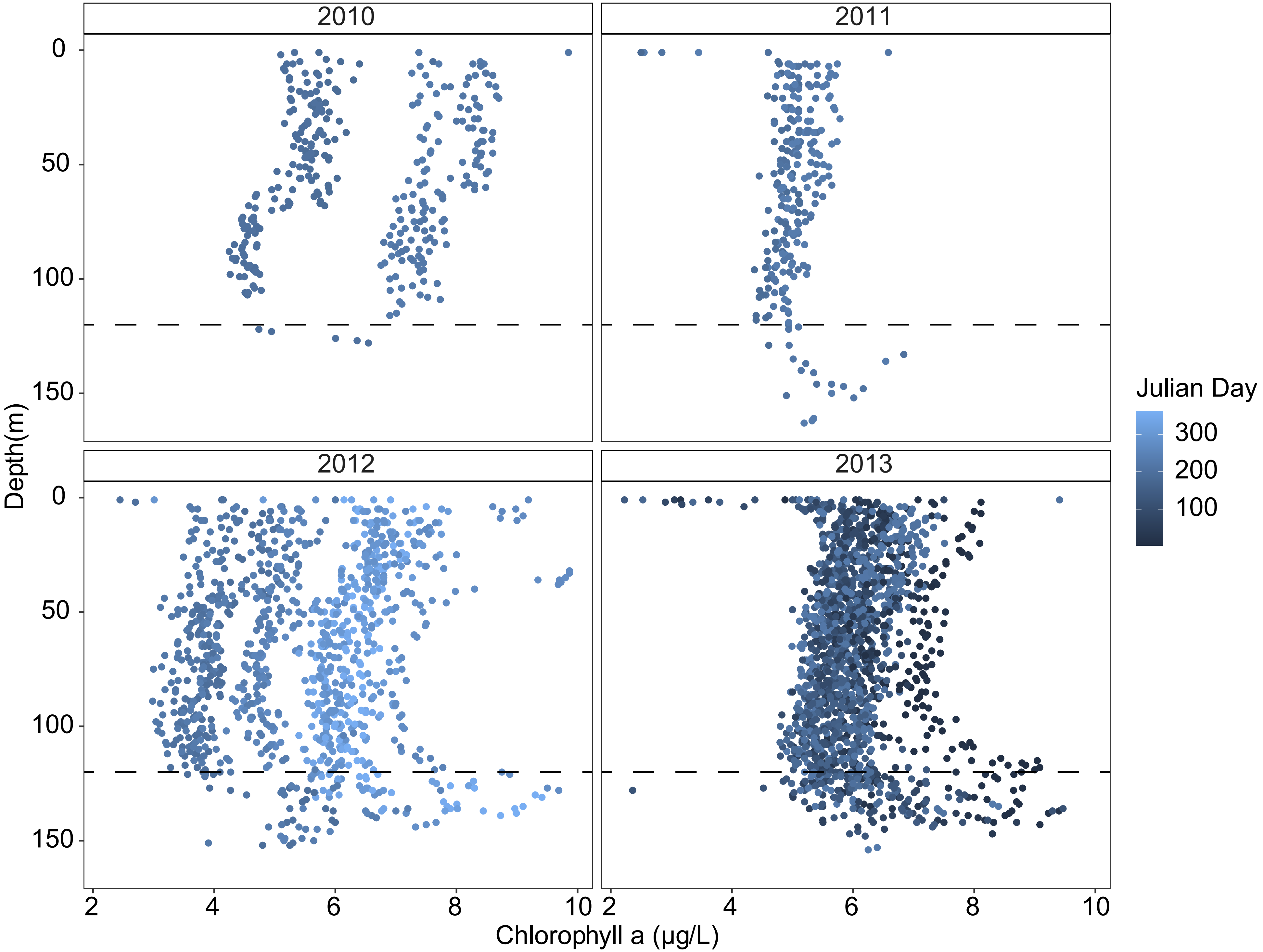

Supplementary Figure 5. Chlorophyll *a* increases with up (down to 150m depth sampling) peak generally around 120m (dashed line). Over one year, chlorophyll *a* increases across all depth (points shift towards the right).
