# Supplementary material for: Depth-discrete metagenomics reveals the roles of microbes in biogeochemical cycling in the tropical freshwater Lake Tanganyika
Source: ISME J. 2021 Feb 9;15(7):1971–86. doi: 10.1038/s41396-021-00898-x (PMC8245535; doi:10.1038/s41396-021-00898-x)
Supplement: Supplementary file 7 — Figure S6 [file 41396_2021_898_MOESM7_ESM.pdf]

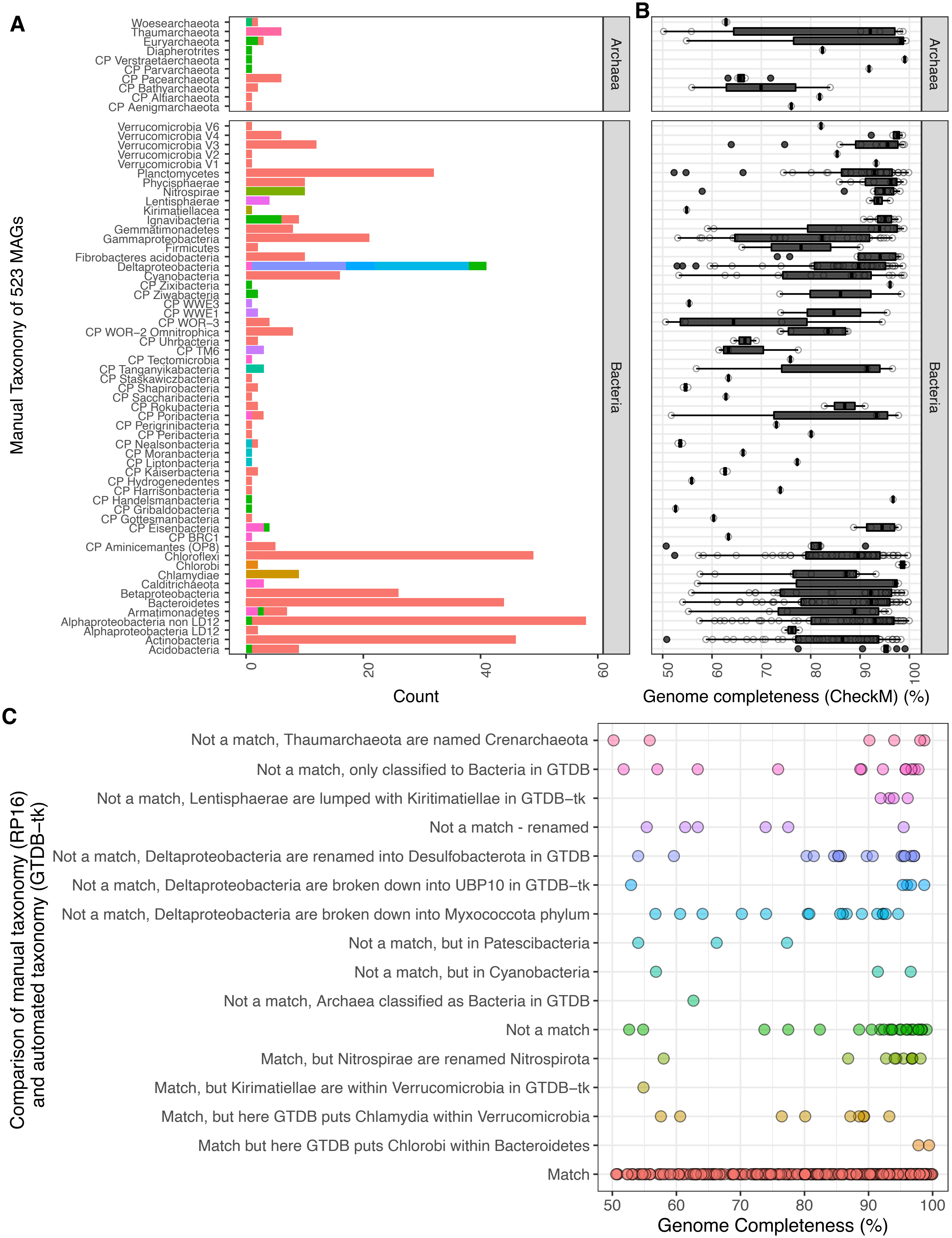

Supplementary Figure 6. A. Comparison of manual RP16 taxonomy versus the GTDB-tk automated taxonomy. **B.** Distribution of completeness levels on for each taxonomy group. **C.** Distribution of completeness values among categories. The colors in this panel is the legend for the colors in panel A. There were matches across the range of completeness values ("Match"). However, when there is a mismatch, it is not always because the genome is less complete.
