# Supplementary material for: Depth-discrete metagenomics reveals the roles of microbes in biogeochemical cycling in the tropical freshwater Lake Tanganyika
Source: ISME J. 2021 Feb 9;15(7):1971–86. doi: 10.1038/s41396-021-00898-x (PMC8245535; doi:10.1038/s41396-021-00898-x)
Supplement: Supplementary file 8 — Figure S7 [file 41396_2021_898_MOESM8_ESM.pdf]

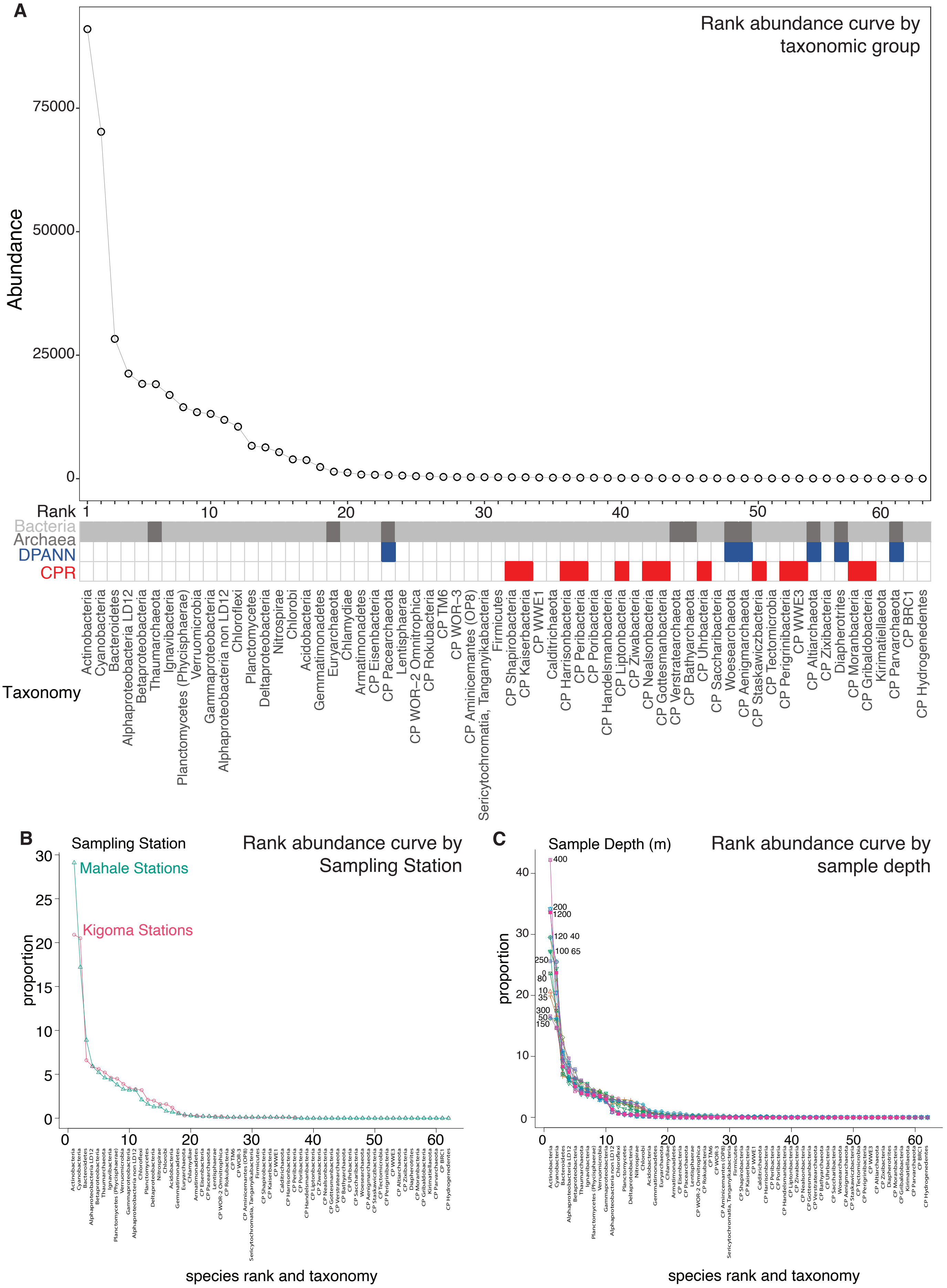

Supplementary Figure 7. A. Rank abundance curve overall for all samples
B. Rank Abundance curve by station C. Rank abundance curve for all samples, grouped by sample depth (meters).
