# Supplementary material for: Depth-discrete metagenomics reveals the roles of microbes in biogeochemical cycling in the tropical freshwater Lake Tanganyika
Source: ISME J. 2021 Feb 9;15(7):1971–86. doi: 10.1038/s41396-021-00898-x (PMC8245535; doi:10.1038/s41396-021-00898-x)
Supplement: Supplementary file 9 — Figure S8 [file 41396_2021_898_MOESM9_ESM.pdf]

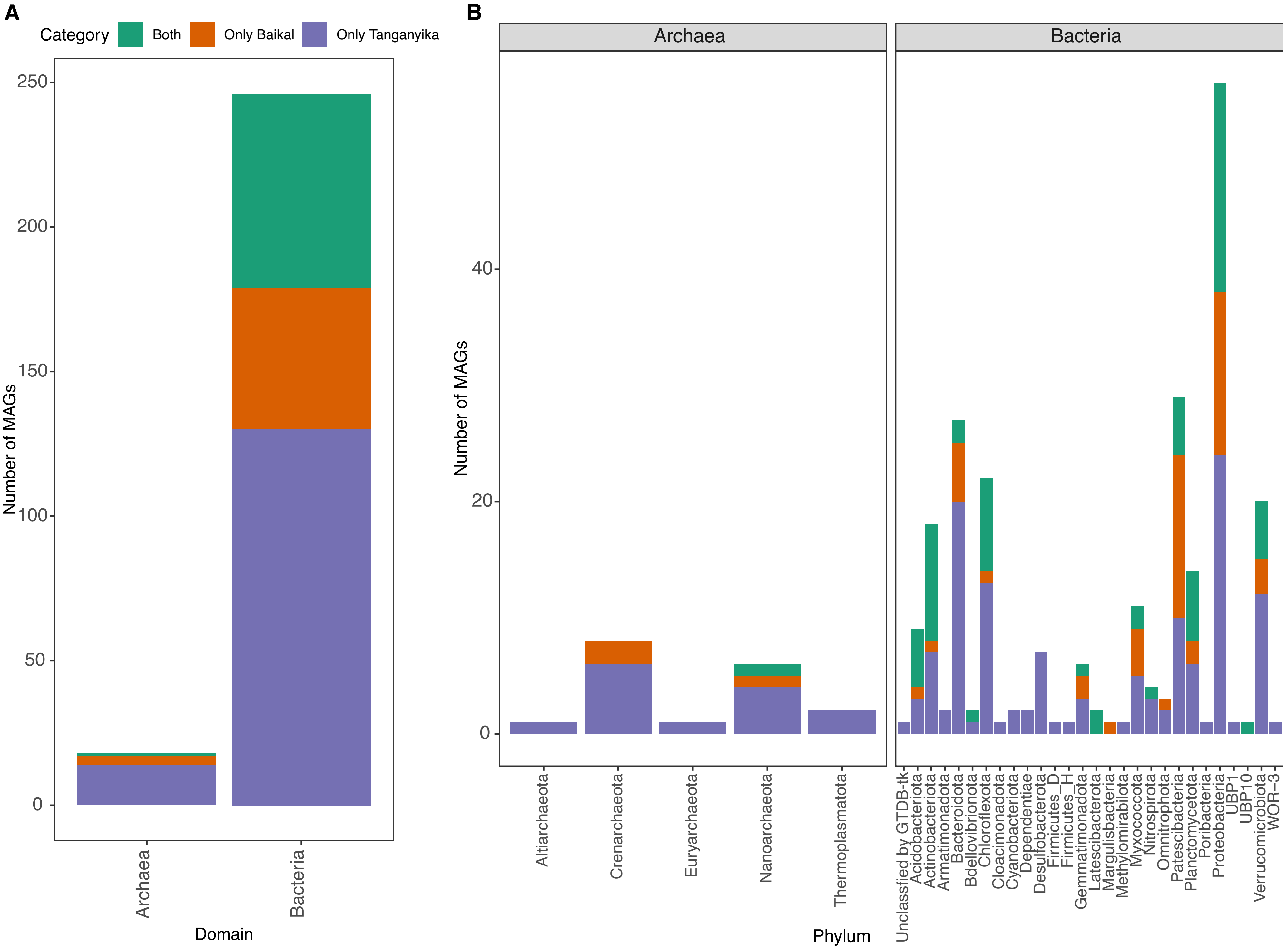

Supplementary Figure 8. Comparison of the taxonomic diversity between Lake Baikal and Lake Tanganyika. A. Number of MAGS shared between the two lakes and unique to either lake. B. Comparison of taxonomic diversity at the phylum-level between the two lakes.
