# Supplementary figures and images for: Depth-discrete metagenomics reveals the roles of microbes in biogeochemical cycling in the tropical freshwater Lake Tanganyika
Source: ISME J. 2021 Feb 9;15(7):1971–86. doi: 10.1038/s41396-021-00898-x (PMC8245535; doi:10.1038/s41396-021-00898-x)

# Sulfur

## 39 taxonomic groups and 361 distinct MAGs

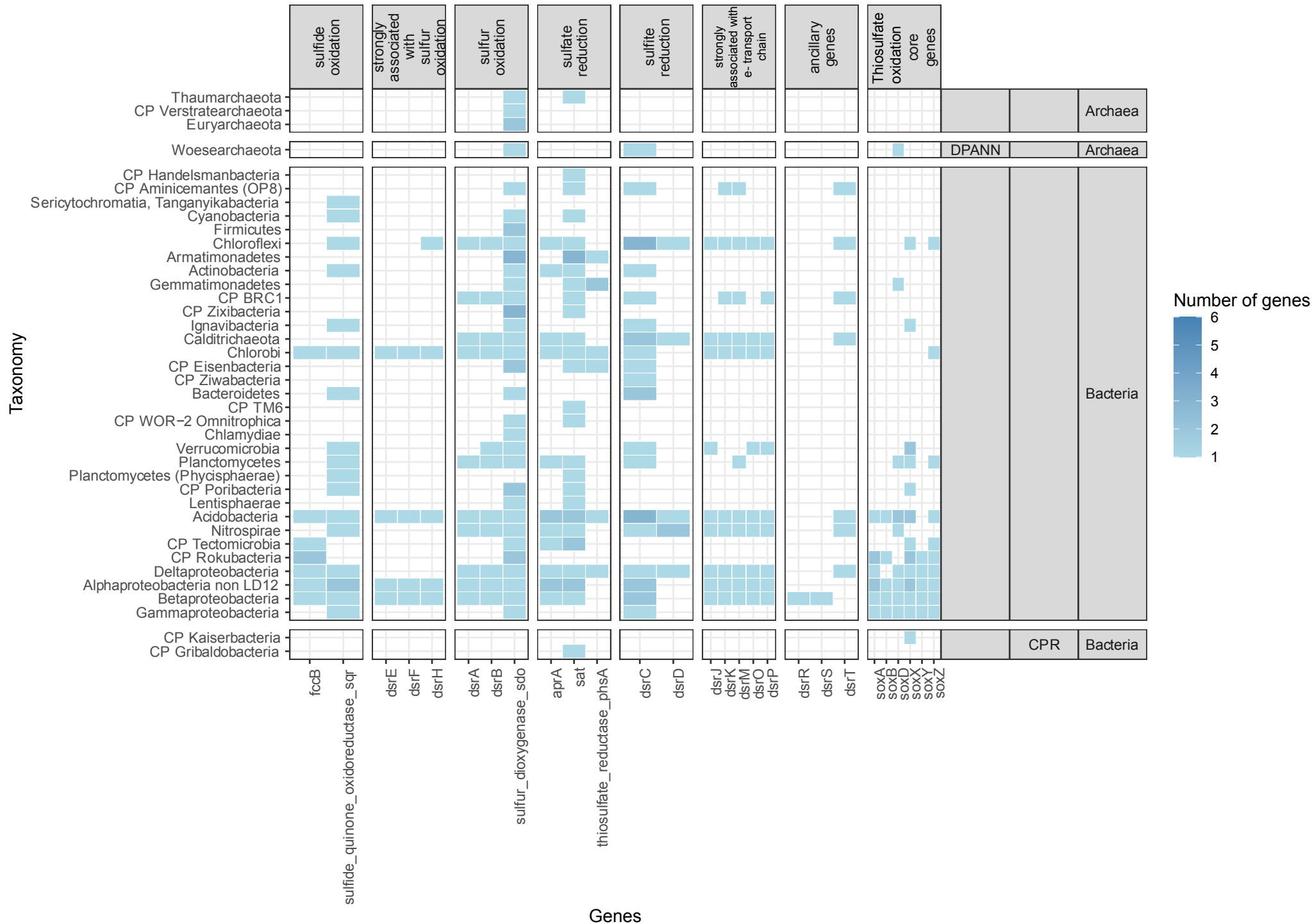

Supplement: Supplementary file 12 — Figure S11 [file 41396_2021_898_MOESM12_ESM.pdf]
