# Supplementary material for: Depth-discrete metagenomics reveals the roles of microbes in biogeochemical cycling in the tropical freshwater Lake Tanganyika
Source: ISME J. 2021 Feb 9;15(7):1971–86. doi: 10.1038/s41396-021-00898-x (PMC8245535; doi:10.1038/s41396-021-00898-x)
Supplement: Supplementary file 15 — Figure S14 [file 41396_2021_898_MOESM15_ESM.pdf]

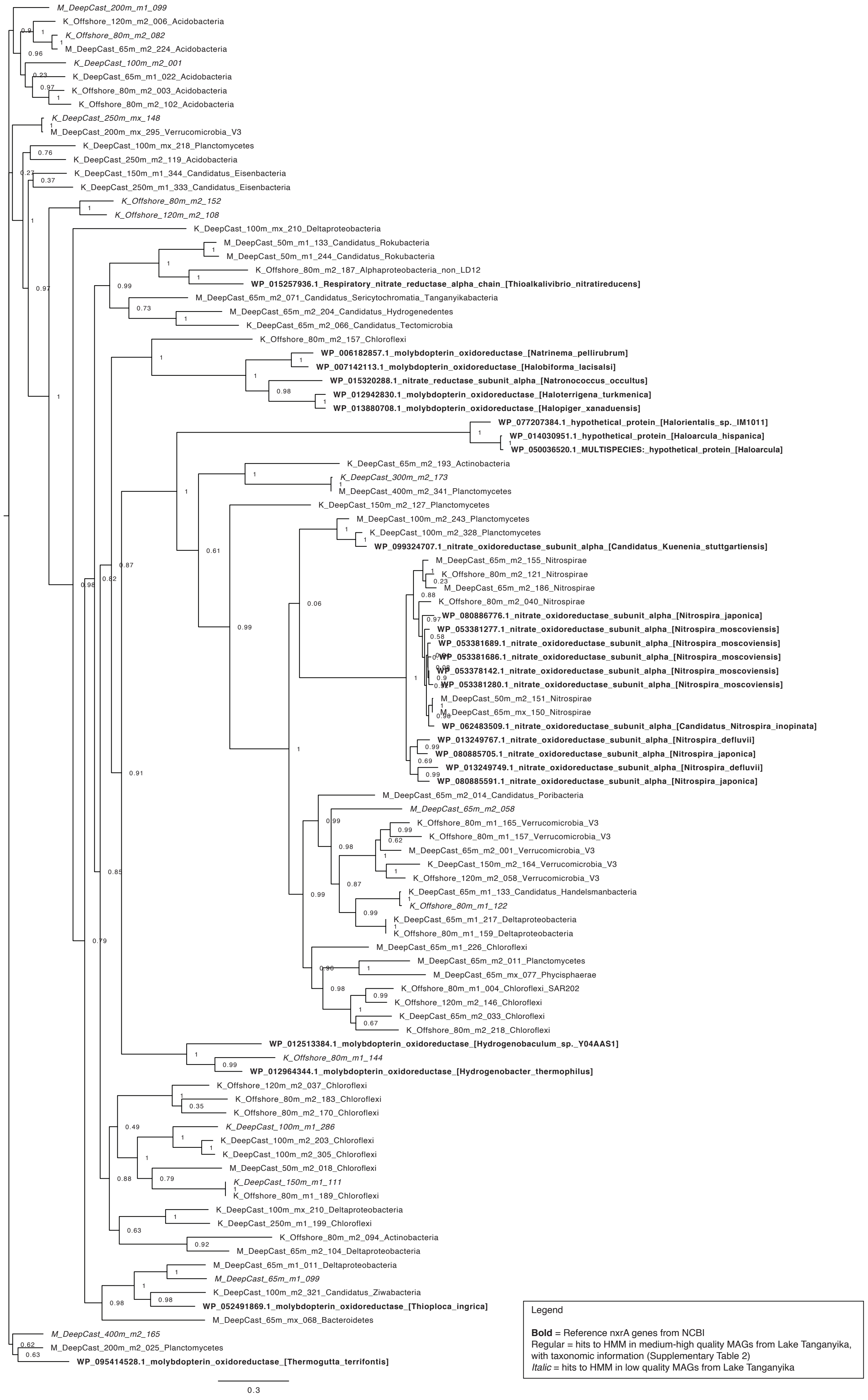

Supplementary Figure 14. Single-gene phylogeny of nxrA to identify (MAFFT, RAxML) different groups of nitrite-oxidizing bacteria or nitrate-reducing bacteria.
